# Transient accumulation of subretinal fluid after half-fluence photodynamic therapy in neovascular age-related macular degeneration

**DOI:** 10.1186/s12886-021-01867-w

**Published:** 2021-02-22

**Authors:** Min Ho Kim, Yoo-Ri Chung, Ji Hun Song

**Affiliations:** 1 Allbarun Eye Clinic, Suwon, Republic of Korea; 2 Department of Ophthalmology, Ajou University School of Medicine, 164 World Cup-ro, Yeongtong-gu, 16499 Suwon, Republic of Korea

**Keywords:** Blood-retinal barrier, Photodynamic therapy, Retinal pigment epithelium

## Abstract

**Background:**

Photodynamic therapy (PDT) is known to occlude choroidal neovascularisation selectively, and there have been several reports on its adverse effects on the normal choroid and retinal pigment epithelium, resulting in decreased vision.

**Methods:**

This retrospective interventional case series aimed to investigate the changes in visual acuity and retinal thickness in the immediate post-treatment period after half-fluence PDT, administered alone or with anti-vascular endothelial growth factor and steroids, in 29 eyes (26 patients) with neovascular age-related macular degeneration. The patients’ best-corrected visual acuity (BCVA) and central foveal thickness (CFT) on optical coherence tomography images were measured 1 day, 1 week, and 1 month post-treatment.

**Results:**

Compared to the pre-treatment CFT (270.38 μm), the mean CFT was significantly increased 1 day post-treatment (387.07 μm, *P* = 0.001), which then started to decrease, with a mean CFT of 269.32 μm (*P* = 0.516) at 1 week, and of 240.66 μm (*P* = 0.066) at 1 month post-treatment. All CFT increases were due to the accumulation of subretinal fluid (SRF), rather than the intraretinal or subretinal pigment epithelium fluid. Relative to the pre-treatment BCVA (0.59 logMAR), the mean BCVA at 1 day (0.74 logMAR, *P* = 0.005) and 1 week (0.75 logMAR, *P* = 0.002) post-treatment was significantly deteriorated; however, it recovered to 0.62 logMAR at 1 month. The patterns of change in CFT and BCVA did not differ according to treatment modality.

**Conclusions:**

Half-fluence PDT resulted in accumulation of SRF in the immediate post-treatment period; this damage mostly recovered within a week, and the BCVA was restored within a month.

**Supplementary Information:**

The online version contains supplementary material available at 10.1186/s12886-021-01867-w.

## Background

Age-related macular degeneration (AMD) is the leading cause of irreversible blindness in the elderly population in developed countries [1]. Severe vision loss often occurs in neovascular AMD with choroidal neovascularisation (CNV) or polypoidal choroidal vasculopathy (PCV) [1]. PCV is a common subtype of exudative AMD in the Asian population [2, 3]. Photodynamic therapy (PDT) has been shown to be effective in the treatment of CNV, especially PCV; however, PDT has limitations that include inadequate vision improvement and the risk of choroidal atrophy [4, 5]. PDT achieves selective regression of CNV due to the selectivity of verteporfin for the endothelium, with the high expression of low-density lipoprotein receptors in the newly developed choroidal vasculature [6]. Although anti-vascular endothelial growth factor (VEGF) agents have become the mainstay for the treatment of CNV [7], there is still a need for PDT, especially in eyes that are refractory to various anti-VEGF agents. Studies have demonstrated that PDT combined with intravitreal injection of an anti-VEGF agent is non-inferior or even superior to monotherapy with PDT or anti-VEGF in eyes with PCV or CNV [8–10].

PDT is known to occlude CNV selectively and safely without damaging the adjacent retina [4, 5]. However, it has been suggested that PDT can cause adverse effects on the normal choroid and retinal pigment epithelium (RPE), resulting in decreased vision, despite successful treatment of CNV [11, 12]. Some studies have reported decreased functioning of the intercellular tight junctions in the RPE cells and vascular endothelial damage in the surrounding physiologic choroid due to PDT, resulting in the dysfunction of the RPE and the overlying photoreceptors [13, 14]. Complications after PDT have also been reported; these range from transient visual impairments due to the accumulation of intraretinal or subretinal fluid (SRF) to permanent vision loss secondary to persistent choroidal atrophy [15–18]. Severe complications after PDT, such as subretinal haemorrhage, supra-choroidal haemorrhage, or RPE tear, can lead to permanent loss of vision [19–22]. Safety-enhanced PDT reduces the risk of these complications by decreasing the fluence rate, duration of light exposure, and dose of verteporfin, particularly in the treatment of chronic central serous chorioretinopathy [23]. The subfoveal choroidal thickness decreases after the half-fluence PDT, along with the resolution of the accumulated SRF in patients with central serous chorioretinopathy, suggesting an effect of PDT on the luminal areas of the choroid [24]. Moreover, safety-enhanced PDT with reduced light seems to be as effective as standard PDT in the treatment of occult CNV [25]. However, neither the transient accumulation of SRF that occurs even after safety-enhanced half-fluence PDT, nor the mechanism underlying the transient fluid collection after PDT has been fully elucidated.

The aim of this study was to investigate the change in visual acuity and retinal thickness in the immediate post-treatment period after safety-enhanced half-fluence PDT, administered alone or with anti-vascular endothelial growth factor and steroids, in eyes with neovascular AMD.

## Methods

This retrospective interventional case series was approved by the Institutional Review Board (IRB) of Ajou University Medical Centre in Suwon, South Korea. This study was conducted in accordance with the tenets of the Declaration of Helsinki and the relevant guidelines and regulations by the IRB of the Ajou University Medical Center. The need for informed consent was waived in view of the retrospective nature of the study by the IRB of the Ajou University Medical Center. Patients aged ≥50 years with subfoveal or juxtafoveal CNV or PCV, who were treated with PDT between June 2007 and December 2017 were included. PDT with verteporfin (Visudyne; Novartis AG, Bulach, Switzerland) was performed using the half-fluence method, i.e., a light fluence rate (300 mW/cm^2^ for 83 s [25 J/cm^2^]) lower than that in the guidelines published by the Treatment of Age-Related Macular Degeneration with Photodynamic Therapy Study Group [5].

The exclusion criteria were as follows: treatment with a standard PDT regimen; treatment with PDT within the previous 6 months; treatment with intravitreal anti-VEGF injection within the previous 3 months; treatment for chronic central serous chorioretinopathy; and severe haemorrhage or large pigment epithelium detachment interfering with interpretation of the optical coherence tomography (OCT) images. PDT with or without anti-VEGF treatment was administered by the same retinal specialist (J.H.S.).

Ophthalmic findings, including best-corrected visual acuity (BCVA) and fundus characteristics, were collected from the patients’ medical records. BCVA, measured as the Snellen visual acuity ratio, was converted to the logarithm of the minimum angle of resolution (logMAR) for the statistical analysis. Fluorescein angiography and indocyanine green angiography were performed during the week before treatment. Serial evaluations using either Stratus OCT (Carl Zeiss Meditec Inc., Dublin, CA, USA) or Spectralis OCT (Heidelberg Engineering, Heidelberg, Germany) were performed within the week before treatment, and then 1 day, 1 week, and 1 month after treatment. The central foveal thickness (CFT) was defined as the thickness from the inner surface of the RPE to the internal limiting membrane. The subfoveal choroidal thickness (SFCT) was defined as the distance from the outer border of the hyper-reflective line corresponding to the RPE perpendicular to the chorioscleral interface. The main outcome measures were the BCVA (logMAR) and CFT recorded 1 week before treatment, and then 1 day, 1 week, and 1 month after treatment. Information concerning any combined treatment, such as intravitreal injection of an anti-VEGF agent (bevacizumab or ranibizumab) or corticosteroids, was also obtained from the patients’ medical records to evaluate the effect of the combined treatment on the main outcomes. Data sets that support the findings of this study are available in Additional file 1.

The statistical analysis was performed using SPSS version 23.0 (SPSS Inc., Armonk, NY, USA). Numerical variables are presented as mean ± standard deviation, whereas categorical variables are presented as counts and proportions. A parametric test is used if the sample size is large enough and the central limit theorem applies. A non-parametric test, such as the Wilcoxon signed-rank test, is used in cases where the central limit theorem does not apply. In this study, the groups were compared using the Wilcoxon signed-rank test for changes in the BCVA and CFT. The Friedman test was used to investigate changes in the choroidal thickness after PDT. Repeated measures ANOVA was used to compare the pattern of changes in the mean values of the BCVA and CFT over time, according to the type of lesion or treatment modality. Spearman’s correlation analysis was performed to investigate factors associated with a change in the CFT. A *P*-value of < 0.05 was considered statistically significant.

## Results

Twenty-nine eyes with subfoveal or juxtafoveal CNV or PCV in 26 patients were enrolled in the study (Table 1). Fourteen eyes had predominantly or minimally classic CNV associated with AMD, 13 had PCV, and two had CNV as a result of retinal angiomatous proliferation (RAP)*.* Seventeen patients (59%) were male and the mean ± standard deviation age was 66.5 ± 13.2 (range, 28–84) years. The mean duration of disease before treatment was 26.3 ± 31.3 (range, 1–132) months. Fifteen eyes (52%) had been treated with PDT for more than 6 months before inclusion in the study; the mean number of treatments in these eyes was 2.5 ± 1.6 (range, 1–6). Twenty-seven eyes (93%) had been treated with anti-VEGF agents for more than 3 months before inclusion; the mean number of injections was 5.8 ± 4.7 (range, 1–16).

**Table 1:** Clinical characteristics of eyes treated with PDT

Included eye	Patient characteristics				Lesion type	Pre-PDT BCVA (logMAR)	Disease duration (months)	Previous PDT sessions(n)	Previous anti-VEGF injections (n)	Pre-PDT RPE atrophy	Treatment methods	Spot size (μm)
	Age (years)	Sex	DM	HTN								
1	81	M	Y	N	CNV	1.00	132	1	8	Y	PDT + IVB	5900
2	52	F	N	N	CNV	0.80	1	0	0	Y	PDT + IVB	3500
3	82	F	N	N	CNV	0.92	108	4	1	Y	PDT + Dexamethasone + IVB	3600
4	66	M	Y	Y	PCV	0.49	16	2	3	Y	PDT + Dexamethasone + IVB	3700
5	84	M	N	N	CNV	0.49	48	5	7	Y	PDT + Dexamethasone + IVB	2900
6	79	F	N	Y	RAP	0.80	1	0	1	Y	PDT + Dexamethasone + IVB	4500
7	78	F	N	Y	PCV	1.30	20	2	7	N	PDT + IVB	5800
8	60	F	N	N	CNV	0.10	68	6	3	Y	PDT + IVB	4100
9	75	F	N	N	PCV	1.30	21	2	1	N	PDT + Dexamethasone + IVB	4900
10	73	M	Y	Y	PCV	0.30	18	1	1	N	PDT + Dexamethasone + IVB	5200
11	71	M	N	Y	PCV	1.30	48	3	1	Y	PDT + IVB	6400
12	65	M	N	N	CNV	0.80	8	0	1	N	PDT	5050
13	60	M	N	N	CNV	0.49	19	3	11	N	PDT + IVR	1900
14	68	F	N	N	RAP	0.00	12	1	1	Y	PDT + IVB	1400
15	62	M	N	Y	CNV	0.80	9	1	2	N	PDT + Dexamethasone + IVB	3200
16	64	M	N	Y	CNV	0.80	26	4	9	N	PDT + IVB	2700
17	52	M	N	N	CNV	0.20	4	1	1	Y	PDT + Dexamethasone + IVB	3200
18	28	M	N	N	CNV	N/A	1	0	1	Y	PDT + IVB	2600
19	50	F	N	N	PCV	0.00	1	0	0	N	PDT + IVR	4800
20	34	M	N	N	CNV	0.10	11	0	3	N	PDT	1900
21	67	M	N	N	PCV	0.70	8	0	9	N	PDT + IVB	3700
22	69	M	N	N	PCV	0.52	22	1	14	N	PDT	4000
23	74	M	N	N	PCV	0.52	17	0	14	N	PDT	1500
24	70	F	N	N	PCV	0.52	14	0	6	N	PDT	2800
25	70	F	N	N	PCV	0.82	14	0	8	N	PDT	3300
26	74	F	Y	Y	PCV	0.70	60	0	9	N	PDT	3700
27	67	F	N	N	CNV	0.10	24	0	9	N	PDT	2500
28	77	M	N	N	PCV	0.00	30	0	16	N	PDT	1500
29	77	M	N	N	CNV	0.70	3	0	10	N	PDT	4200

*BCVA* best-corrected visual acuity, *CNV* choroidal neovascularisation, *DM* diabetes mellitus, *F* female, *HTN* hypertension, *IVB* intravitreal injection of bevacizumab, *IVR* intravitreal injection of ranibizumab, *logMAR* logarithm of the minimum angle of resolution, *M* male, *N* no, *N/A* not accessed, *PCV* polypoidal choroidal vasculopathy, *PDT* photodynamic therapy, *RAP* retinal angiomatous proliferation, *RPE* retinal pigment epithelium, *VEGF* vascular endothelial growth factor, *Y* yes

Ten eyes had received PDT as monotherapy and 19 had received PDT combined with intravitreal injections. Nine eyes in the combination therapy group had received PDT with bevacizumab (1.25 mg/0.05 mL), two had received ranibizumab (0.5 mg/0.05 mL), and eight had received bevacizumab (1.25 mg/0.05 mL) and dexamethasone (400 μg) on the same day as PDT.

**Fig. 1:**
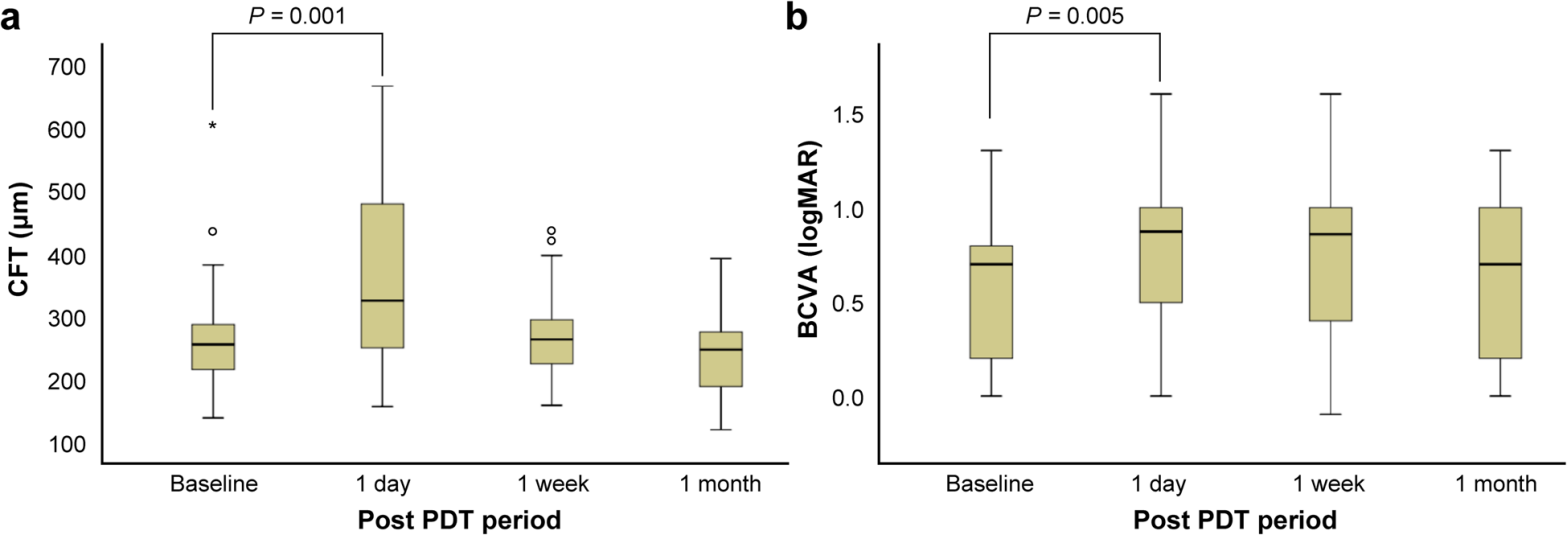
Changes in the mean CFT and BCVA after PDT. **a** A significant mean increase in CFT was observed 1 day after PDT compared to baseline (*P* = 0.001, Wilcoxon signed-rank test), followed by spontaneous decreases at 1 week and 1 month after treatment. **b** A significant deterioration in the BCVA was noted on the day after PDT compared to baseline (*P* = 0.005, Wilcoxon signed-rank test), but the BCVA improved spontaneously by 1 month after treatment. The error bars represent the distance of 1.5 times the interquartile range (IQR) below the 1st quartile and above the 3rd quartile. For outliers, the cases exceeding 1.5 times the IQR above the 3rd quartile are presented as a circle (°), and one case exceeding 3 times the IQR above the 3rd quartile is presented as an asterisk (*). BCVA, best-corrected visual acuity; CFT, central foveal thickness; logMAR, logarithm of the minimum angle of resolution; PDT, photodynamic therapy

Serial OCT images revealed a significant increase in the mean CFT on day 1 after PDT (270.38 ± 87.78 μm vs. 387.07 ± 190.67 μm; *P* = 0.001, Wilcoxon signed-rank test). However, a spontaneous decrease in the mean CFT to a level similar to that noted before the PDT was observed at 1 week (269.32 ± 70.23 μm, *P* = 0.516, Wilcoxon signed-rank test) and 1 month (240.66 ± 60.45 μm, *P* = 0.066, Wilcoxon signed-rank test) after treatment (Fig. 1a).

**Fig. 2:**
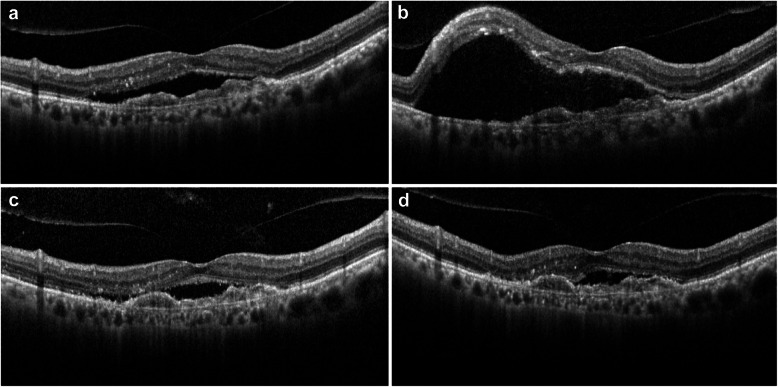
Representative serial OCT findings for study eye number 29. **a** An OCT image acquired before PDT showing a mild collection of SRF (448 μm). **b** On the day after PDT, there was an increase in central foveal thickness (565 μm) that was mainly due to the accumulation of SRF. **c** The accumulated SRF was spontaneously absorbed at 1 week after PDT (393 μm). **d** An OCT image obtained 1 month after PDT shows complete resolution of the accumulated SRF. OCT, optical coherence tomography; PDT, photodynamic therapy; SRF, subretinal fluid

Figure 2 shows representative OCT findings for a patient with accumulation of SRF immediately after PDT and the subsequent changes that occurred over time.

The mean increase in the CFT for all eyes on the day following PDT (116.69 ± 192.98 μm) was due to accumulation of SRF rather than the collection of intraretinal or sub-RPE fluid. Twenty-one (72%) of the 29 eyes showed an increase in the CFT on the day following PDT, while eight did not. By 1 month, 20 (95%) of the 21 eyes showed a spontaneous decrease in the CFT, compared with the value obtained the day after the treatment. There was no association between the change in CFT on the day after treatment and age (*P* = 0.464, Spearman’s correlation test), duration of symptoms (*P* = 0.489, Spearman’s correlation test), number of previous PDT sessions (*P* = 0.726, Spearman’s correlation test), size of the PDT spot (*P* = 0.785, Spearman’s correlation test), or previous atrophy of the RPE (*P* = 0.740, Spearman’s correlation test).

SFCT was measurable in seven eyes, which were examined by enhanced depth imaging spectral-domain OCT. There was no significant change in SFCT from the baseline to any of the time-points examined in the study following PDT (244.86 ± 75.09 μm at baseline; 209.86 ± 34.32 μm 1 day after PDT; 240.00 ± 74.93 μm at 1 week; 268.86 ± 71.53 μm at 1 month; *P* = 0.156, Friedman test).

The BCVA deteriorated significantly from before the PDT (0.59 ± 0.39 logMAR) to 1 day and 1 week after the PDT (0.74 ± 0.42 logMAR, *P* = 0.005; and 0.75 ± 0.46 logMAR, *P* = 0.002, respectively; Wilcoxon signed-rank test), and improved spontaneously to a level similar to that before the PDT at 1 month after the treatment (0.62 ± 0.41, *P* = 0.456, Wilcoxon signed-rank test, Fig. 1b). The BCVA subsequently recovered to the CFT; after 1 month, 23 eyes (79%) showed spontaneous BCVA improvement, compared with that measured on the day after treatment, while 6 (21%) still exhibited a deterioration in BCVA.

**Fig. 3:**
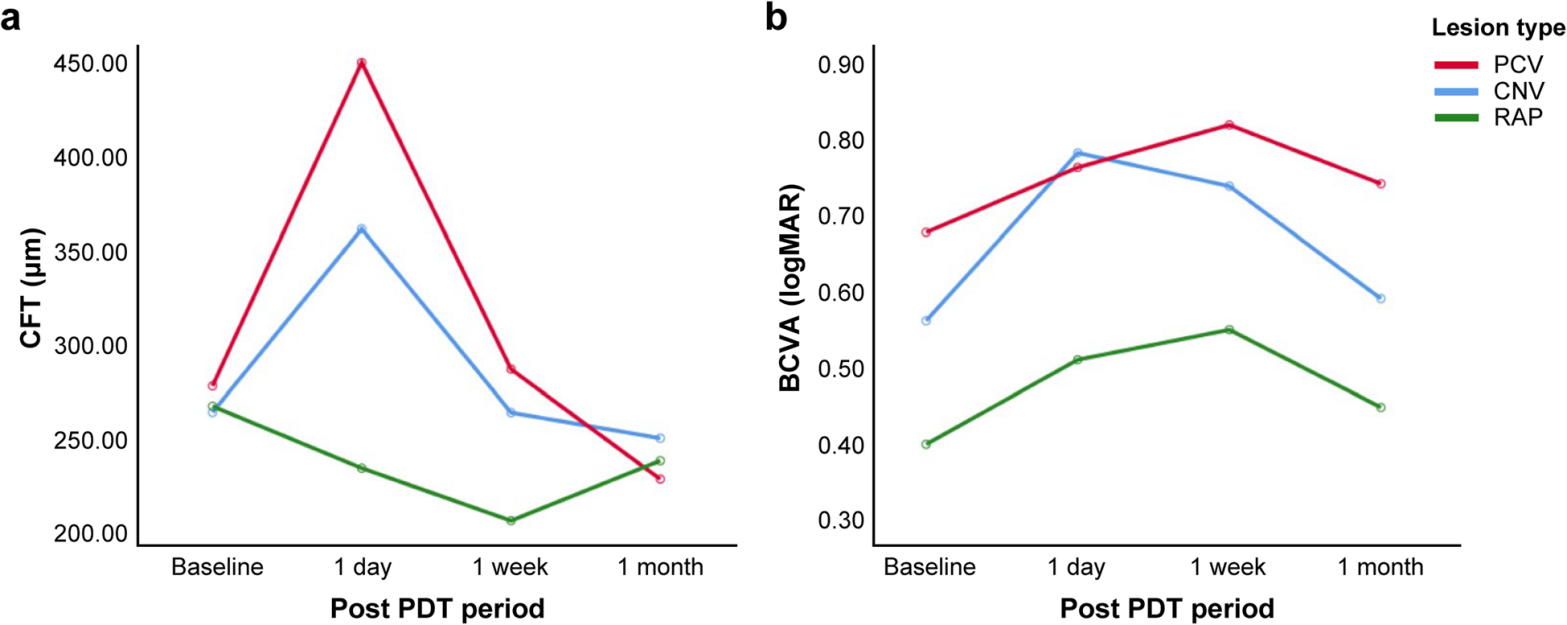
Changes in the mean CFT and BCVA according to the type of lesion. The red, blue, and green lines represent the patient subgroups with PCV, CNV other than PCV, and RAP, respectively. For the multiple comparison, repeated measures ANOVA was used to compare the pattern of changes in the mean values of the CFT and BCVA at the baseline, and then 1 day, 1 week and 1 month after treatment, according to the type of lesion. **a** The pattern of change in the CFT over time was slightly but not significantly different between the RAP group and the other groups (*P* = 0.395, repeated measures ANOVA). **b** The pattern of change in the BCVA over time was similar for the different types of lesions (*P* = 0.679, repeated measures ANOVA). BCVA, best-corrected visual acuity; CFT, central foveal thickness; CNV, choroidal neovascularisation; logMAR, logarithm of the minimum angle of resolution; PCV, polypoidal choroidal vasculopathy; PDT, photodynamic therapy; RAP, retinal angiomatous proliferation

There was no significant difference in the pattern of mean change in the CFT over time, according to the type of lesion (*P* = 0.395, repeated measures ANOVA, Fig. 3a). One of the two patients with RAP showed a decrease in the CFT on the day after PDT, suggesting that the pattern of change in the CFT in eyes with RAP was different from that in the other groups; however, the difference was not statistically significant. There was also no statistically significant difference in the pattern of change in the BCVA according to the type of lesion among the three groups (*P* = 0.679, repeated measures ANOVA, Fig. 3b).

**Fig. 4:**
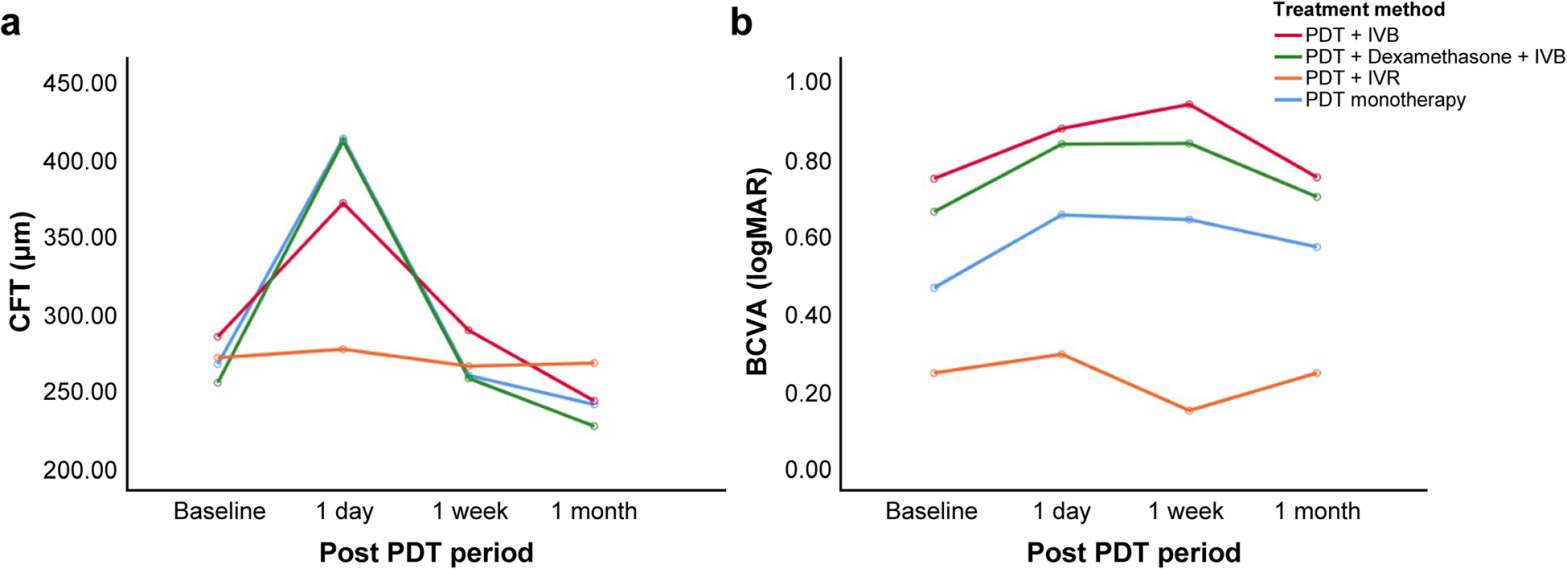
Changes in the mean CFT and BCVA according to the treatment modality. The red, green, orange, and blue lines represent the patient subgroups treated with PDT and IVB; PDT, steroid, and IVB; PDT and IVR; and PDT alone, respectively. For the multiple comparison, repeated measures ANOVA was used to compare the pattern of changes in the mean values of the CFT and BCVA at baseline, and then 1 day, 1 week and 1 month after treatment, according to the treatment modality. **a** The pattern of change in the CFT over time was similar among the treatment modalities (*P* = 0.974, repeated measures ANOVA). **b** The pattern of change in the BCVA over time was also similar among the treatment modalities (*P* = 0.247, repeated measures ANOVA). BCVA, best-corrected visual acuity; CFT, central foveal thickness; IVB, intravitreal injection of bevacizumab; IVR, intravitreal injection of ranibizumab; logMAR, logarithm of the minimum angle of resolution; PDT, photodynamic therapy

The patterns of change in the CFT and BCVA did not differ according to the treatment modality. Eyes treated with both PDT and intravitreal ranibizumab (two patients) tended to exhibit a smaller increment in the CFT 1 day after the treatment, compared with the other two treatment groups; however, the pattern of change was not statistically significant (*P* = 0.974, repeated measures ANOVA, Fig. 4a). This subgroup also showed an earlier recovery of the BCVA, compared with the other groups; the difference was not statistically significant (*P* = 0.247, repeated measures ANOVA, Fig. 4b).

## Discussion

PDT is a well-established treatment for CNV in exudative AMD as well as for PCV [2, 26]. There have been several reports on transient serous retinal detachment after PDT [15, 16]. The serial OCT scans in our study showed accumulation of SRF 1 day after the PDT, which started to resolve 1 week later. Visual deterioration was noted at the same time as the foveal changes, the recovery of which was slower than the resolution of the accumulation of SRF. Accumulation of SRF might have been the main contributor to the decreased vision because the pattern of change in visual acuity corresponded to the pattern of change in SRF on OCT images, despite the slight time lag. Mennel et al. [18] similarly reported transient neurosensory retinal detachment after PDT monotherapy in six patients with classic CNV and four with occult CNV, which occurred 2 days after PDT and resolved spontaneously by 1 week post-treatment. Our study revealed that transient accumulation of SRF can develop even after safety-enhanced half-fluence PDT, which was used to reduce the adverse effects of standard PDT.

There are several possible explanations for the transient fluid collection that occurs in the subretinal space after PDT. PDT may damage the RPE cells or their intercellular tight junctions (zonulae occludentes), resulting in a change in the outer blood-retinal barrier (BRB) [12]. Decreased functioning of the intercellular tight junctions by a therapeutic level of PDT, without permanent damage to RPE cells, has been reported in an in vitro model [13]. In that study, no swelling or structural alterations were observed in the RPE; however, subtle intercellular blisters with a normal junctional complex were noted [13]. Transient accumulation of SRF can be considered a clinical sign of functional rather than anatomic breakdown of the outer BRB [13]. In this context, decreased functioning of the RPE might initially lead to immediate accumulation of SRF after half-fluence PDT; in our study, this accumulation resolved spontaneously as the RPE recovered over time. Vascular endothelial damage in the surrounding physiologic choroid, resulting in ischaemic damage to the overlying RPE, has also been suggested as an explanation for the initial dysfunction of the RPE caused by PDT [14]. Spontaneous recovery of the impaired choroid circulation following PDT has also been reported [27], and might explain our finding of a transient change in SRF after PDT and its subsequent recovery. However, perfusion defects within the adjacent choroid starting as early as 1 day after PDT and persisting for several months have also been demonstrated with standard PDT [28], which is in contrast to the results we obtained with half-fluence PDT.

A less likely explanation is that PDT-induced early direct vascular leakage from the CNV itself can result in accumulation of SRF. The hyperfluorescence seen on fluorescein angiography images was considered equivalent to the increased leakage from CNV itself 1 day after the PDT, probably as a result of damage to the vascular endothelium [27]. However, PDT is known to exert its therapeutic effects by photothrombosis of a neovascular lesion, resulting in decreased choroidal circulation rather than increased vascular leakage [4]. The absence of sub-RPE fluid on the day after PDT also indicates a low possibility of CNV leakage. However, we could not confirm a correlation between the size of the PDT laser spot and the extent of accumulation of SRF.

The comparison between the therapeutic effects of PDT alone and PDT combined with anti-VEGF and a steroid might provide valuable insights into the development of SRF. The levels of VEGF may increase in the early post-treatment period after PDT. A decrease in choroidal circulation can cause ischaemic damage to the overlying outer retina and choroid, resulting in an increased secretion of VEGF and vascular leakage. Therefore, transient accumulation of SRF may be associated with an increased secretion of VEGF and vascular leakage after PDT [11]. If increased levels of VEGF play a prominent role in the accumulation of SRF after PDT, anti-VEGF treatment administered in combination with PDT should have reduced the SRF accumulation. However, in this study, there was no difference in the time course of SRF accumulation between the eyes in which PDT was administered alone and those that received PDT combined with an anti-VEGF agent. Therefore, an increased level of VEGF might not be directly associated with the transient accumulation of SRF after PDT. Furthermore, CNV occlusion is due to photothrombosis that occurs 1 day after PDT, whereas occlusion in the surrounding normal choroid occurs 1 week after PDT [27]. If upregulation of VEGF caused by this ischaemia results in SRF, there should be more accumulation after 1 week than on the day after PDT.

An acute inflammatory reaction after PDT can also induce transient accumulation of SRF. Rogers et al. [29] reported finding an increment in vascular leakage in the treated area immediately after PDT on fluorescein angiography images and suggested that the accumulation of SRF was caused by an acute inflammatory reaction to PDT. Free radicals, reactive singlet oxygen, and inflammatory cytokines induced by PDT can damage the CNV and surrounding physiological cells, resulting in an inflammatory reaction [30]. Dexamethasone, a short-acting steroid with a strong anti-inflammatory effect, has been used to treat macular oedema associated with various retinal disorders [31]. However, the effects of PDT combined with dexamethasone were not significantly different from those of PDT alone in this study, suggesting that inflammation may not be the main factor involved in the immediate accumulation of SRF. Furthermore, there were no significant changes in the choroidal thickness at any of the time-points in the study, which suggests that the accumulation of SRF may be affected by an impaired RPE barrier function rather than by any choroidal inflammation induced by PDT.

In spite of the aforementioned hypotheses, the exact mechanism explaining the transient accumulation of SRF after PDT has not been elucidated. Our study showed that a transient collection of SRF developed in neovascular AMD following half-fluence PDT, which induced less ischaemic and more limited upregulation of anti-VEGF and inflammatory cytokines than standard PDT. Furthermore, patients treated with half-fluence PDT alone and those treated with PDT in combination with anti-VEGF and steroid therapy did not show significant differences in terms of the mean change in CFT. The results of our study strongly suggest that transient damage to RPE function is likely to be responsible for this change; to the best of our knowledge, this is the first in vivo study to reveal that the mechanism underlying the accumulation of SRF after PDT in neovascular AMD, and its effect on the breakdown of outer BRB function. Therefore, caution is required in patients with pre-existing RPE dysfunction. Elderly patients, those with pre-existing RPE atrophy, and those with a history of multiple rounds of PDT might be at a higher risk of severe immediate accumulation of SRF after PDT. Although we did not identify any factors significantly associated with accumulation of SRF in this study, potentially due to the small number of patients in the series, the treatment protocol may need to be individualised according to possible risk factors, such as age, degree of RPE atrophy, and any history of previous PDT, to reduce the risk of accumulation of SRF after PDT and the subsequent poor visual outcome. Both ophthalmologists and patients should be aware of the possibility of a transient deterioration of vision due to accumulation of SRF, even after safety-enhanced PDT.

This study had several strengths, namely the inclusion of a large number of eyes treated with half-fluence PDT and the use of detailed serial examinations (from 1 day to 1 month after treatment). The study also has several limitations, which are mostly related to its retrospective design, in that no additional work-up for decreased vision other than OCT could be performed. Moreover, the combined treatment did not include aflibercept; therefore, its effects could not be evaluated. Finally, the fovea was examined by Stratus OCT in some patients rather than by spectral-domain OCT; therefore, the ability to visualise the fovea in these eyes was limited due to the image quality.

In conclusion, our findings support the notion that half-fluence PDT results in a breakdown of the outer BRB in the immediate post-treatment period. This can cause transient accumulation of SRF in association with transient visual impairments, even if PDT is performed in combination with intravitreal anti-VEGF and steroid injection simultaneously. Most of this damage resolves within a week, and the BCVA is restored within a month. A further study in a larger number of patients is needed to evaluate the factors related to PDT that may affect the function of the RPE and to establish a safe PDT protocol for patients suspected of having decreased RPE function.

## Supplementary Information

**Additional file 1.** Changes in central foveal thickness and best-corrected visual acuity. Dataset that supports the findings of this study.

## Data Availability

The dataset supporting the conclusions of this article is included within the article and its additional file.

## Abbreviations

AMD: age-related macular degeneration
BCVA: best-corrected visual acuity
BRB: blood-retinal barrier
CFT: central foveal thickness
CNV: choroidal neovascularisation
IRB: Institutional Review Board
OCT: optical coherence tomography
PCV: polypoidal choroidal vasculopathy
PDT: photodynamic therapy
RAP: retinal angiomatous proliferation
RPE: retinal pigment epithelium
SFCT: subfoveal choroidal thickness
SRF: subretinal fluid
VEGF: vascular endothelial growth factor
